# Changing how the third 95 is counted: suitable indicators for measuring U = U with findings from Taiwan

**DOI:** 10.1186/s12981-024-00626-3

**Published:** 2024-06-20

**Authors:** Hsun-Yin Huang, Yu-Ching Huang, Hsiu-Yun Lo, Pei-Chun Chan, Chia-Chi Lee

**Affiliations:** 1 Division of Chronic Infectious Diseases, Centers for Diseases Control, No. 6 Linsen South Road, Taipei, 100 Taiwan; 2 National Research Institute of Chinese Medicine, Ministry of Health and Welfare, Taipei, Taiwan; 3 Institute of Epidemiology and Preventive Medicine, National Taiwan University College of Public Health, Taipei, Taiwan

**Keywords:** Viral suppression, Viral load monitoring, HIV care continuum

## Abstract

**Introduction:**

The World Health Organisation has implemented multiple HIV prevention policies and strived to achieve the 90-90-90 goal by 2020, achieving the 95-95-95 goal by 2030, which refers to 95% of patients living with HIV knowing their HIV status, 95% of patients living with HIV receiving continual care and medication, and 95% of patients living with HIV exhibiting viral suppression. However, how to measure the status of viral suppression varies, and it is hard to indicate the quality of HIV care. The study aimed to examine the long-term viral load suppression in these cases and explore potential factors affecting the control of long-term viral load.

**Methods:**

This study analyzed viral load testing data from HIV patients who are still alive during the period from notification up to 2019–2020. Three indicators were calculated, including durable viral suppression, Viremia copy-years, and Viral load > 1,500 copies/ml, to assess the differences between them.

**Results:**

Among the 27,706 cases included in the study, the proportion of persistent viral load suppression was 87%, with 4% having viral loads exceeding 1,500 copies/ml. The average duration from notification to viral load suppression was 154 days, and the geometric mean of annual viral replication was 90 copies*years/ml. Regarding the last available viral load measurement, 96% of cases had an undetectable viral load. However, we observed that 9.3% of cases, while having an undetectable viral load for their last measurement, did not show consistent long-term viral load suppression. An analysis of factors associated with non-persistent viral load suppression revealed higher risk in younger age groups, individuals with an educational level of high school or below, injection drug users, cases from the eastern region, those seeking care at regional hospitals, cases with drug resistance data, individuals with lower healthcare continuity, and those with an initial CD4 count below 350 during the study period.

**Conclusions:**

The recommendation is to combine it with the indicator of sustained viral load suppression for a more accurate assessment of the risk of HIV transmission within the infected community.

## Introduction

According to the Joint United Nations Programme on HIV/AIDS, approximately 37.7 million surviving cases and 1.5 million new cases of HIV were reported in 2020 [1]. Consequently, HIV/AIDS has become a focus of worldwide efforts in infectious disease prevention. Since 2010, the World Health Organisation has implemented multiple HIV prevention policies to achieve the 90-90-90 goal by 2020. This goal entails ensuring that 90% of patients living with HIV are aware of their HIV status, 90% receive medication, and 90% achieve viral suppression [2], to realise the “Getting to Zero” vision - zero new infections, zero AIDS-related deaths, and zero discrimination, to eradicate HIV/AIDS by 2030 [3].

In 1984, Taiwan reported its first case of HIV. In 2005, Taiwan reported a total of 3,378 HIV infections, representing an increase of 122%, due to injection drug users (IDUs) sharing needles. Then, the government launched a harm reduction programme in 2007 and effectively reduced the number of HIV infections. A total of 1,800–2,300 HIV infections have been reported in recent year, and the majority were due to condomless sex between men who have sex with men (MSM). As of August 2023, Taiwan has accumulated 43,967 cases of HIV and 35,424 patients living with HIV [4].

Quality of HIV care is affected by immediacy of diagnosis, connection to a healthcare system, retention in HIV care, and patient compliance with antiviral medication. According to the study done by US Centers for Disease Control and Prevention, 86% of persons with HIV aware of their infection, 74% of persons with diagnosed infection in care, and 83% of persons in care with viral suppression in 2016 [5]. Suppressing viral replication is a crucial indicator that predicts the compliance of patients towards their antiviral medication and treatment. Continual care helps suppress patients viral load, thereby mitigating their risk of comorbidities, mortality rate, and risk of transmitting the virus to others [6–9].

Amounts of evidence has indicated that if patients with HIV receive regular antiviral treatment for six months or more, the viral load in their blood becomes undetectable, and their ability to transmit the virus through sexual contact diminishes; this gives rise to the concept of undetectable = untransmittable (U = U) [10, 11]. Cohen et al. discovered that serodiscordant couples who received antiviral treatment for HIV exhibited a 96% reduced risk of viral transmission, also reported that couples who had undetectable viral loads did not experience viral transmission [12, 13]. The results indicated that when the viral load was smaller than 200 copies/ml, the number of HIV infections caused by sexual transmission was zero [14, 15]. Suppressing viral replication may serve as a predictor of patient compliance towards antiviral medication. It may also help determine their transmission risk, and serve as an indicator of the outcomes of HIV care.

Clinical research commonly defines viral suppression as a final viral load of less than 200 copies/ml over the preceding 12 months [16, 17]. However, this definition pertains to the detection of viral load at a single time point and does not reflect the long-term changes in viral load. Therefore, certain studies have started monitoring long-term changes in viral load [18, 19]. According to previous studies, the viral load at a single time point may result in the overestimation of stable viral suppression in patients with HIV by approximately 16% [20, 21]. Therefore, long-term monitoring of viral load may help clarify the viral replication pattern of patients over time and may serve as a biological indicator of how the immune system is affected. To predict the rate of mortality, risk of comorbidity, and risk of HIV transmission among patients with HIV, the mean number of viremia copy-years can calculated by averaging each viremia copy-year [18].

In the past, we assessed medication adherence and care condition in HIV patients through a single time point viral load evaluation. Recognizing the necessity for prolonged HIV treatment, we incorporated additional indicators from Crepaz et al.‘s study [21]. The three indicators include sustained durable viral suppression, viremia copy-years, and viral load exceeding 1,500 copies/ml. In this study, we applied these indicators to assess long-term viral suppression in our HIV patients. The results not only facilitate international comparisons but also offer valuable insights into long-term viral load control in Taiwan. This evaluation informs potential management indicators for future routine monitoring. Additionally, factors influencing long-term viral load changes, assessing the quality of medical care for infected individuals, are computed in this study.

## Methods

### Study setting

We used the HIV case management system database maintained by Taiwan CDC to conduct this study. HIV is categorized as notifiable diseases in Taiwan, requiring hospitals to report cases through the notifiable diseases system. Subsequently, the data is transmitted to the HIV case management system, facilitating follow-up actions by public health workers and case managers in designated hospitals. According to HIV/AIDS Prevention and Control Manual, public health workers conduct periodic follow-ups with HIV patients, at least once every three months. During these visits, patients need to receive viral load testing and CD4 test every 3–6 months, public health workers review patients’ medical records and testing data, including CD4 and viral load information transferred by HIV-designated hospitals. Medical record data were collected from the National Health Insurance outpatient and hospitalization database for the period between 2018 and 2020.

### Study design

This study was a retrospective cohort study conduct in the year 2020. The data was population-based, and a total of 37,890 Taiwanese patients with HIV whose infection was reported around Taiwan before 2018 were included in the study. To calculate the long-term viral load control indicator, which necessitates a minimum of 2 years of viral load data, the study includes individuals reported as HIV-infected up to the year 2018 (inclusive). A total of 9,331 patients who lacked viral load data between 2018 and 2020 (6,712 deceased) and 853 patients who underwent only one viral load test (172 deceased) were excluded. The total number of patients included in the final analysis was 27,706.

### The outcome of interest

Three indicators referenced in a study by Crepaz et al. [21] are calculated as follows.

#### Durable viral suppression

To maintain consistency with the common indicators of viral load (VL), viral suppression was defined in this study as a viral load smaller than 200 copies/ml. Durable viral suppression was defined as a patient having at least 2 VL measurements, and all VL values < 200 copies/ml in both 2019 and 2020.

#### Viremia copy-years

The concept of viremia copy-years is similar to that of disease burden. Calculating viremia copy-years may reveal the cumulative burden of HIV on a patient during the observation period [11]. Viremia copy-years are calculated as follows:

$${\text{k}}_{i}\left({J}_{i}\right)=\sum _{j=1}^{{J}_{i}}\left[{t}_{i}\left(j\right)-{t}_{i}(j-1)\right]\times \left[{V}_{i}\left(j\right)+{V}_{i}(j-1)\right]/2$$

where ${\text{k}}_{i}\left({J}_{i}\right)$represents the viremia copy-years for patient *i*, ${t}_{i}\left(j\right)$is the times of viral load measurements after seroconversion, and V_*i*_(*j*) is the viral load of patient *i* at time *j*.

To evaluate patients who underwent two or more viral load tests from 2019 to 2020, the time interval between every two viral load tests was first calculated, and then the mean of the two viral load tests was multiplied by the time interval to obtain the HIV burden. Subsequently, the HIV burden of each patient was summed to calculate their viremia copy-years over the two-year period. Because of the skewed distribution of viremia copy-years, a geometric mean was used instead of an arithmetic mean.

#### Viral load > 1,500 copies/ml

All calculations were performed in accordance with Marks et al. [20], we calculated the number of days with each pair of viral load results above 1,500 copies/ml, summed these estimated days for each patient, and then aggregated the total person-time above the 1,500 threshold over the 2-year period. If two consecutive viral load results changed from below 1,500 to above 1,500 copies/ml, or vice versa, we assessed the number of days above the threshold by calculating the range between the two VL results and determining the relative position of the 1,500 thresholds within that range.

### Statistical analysis procedures

SAS 9.4 (SAS Institute Inc., Cary, Nc, USA) were used for data management and data analysis. Interrupted care was defined as undergoing two consecutive viral load tests separated by a time interval of 12 months or more from 2019 to 2020. Continual care was defined as receiving two or more medical treatments every year, with 90 days or more between every two consecutive treatments. Sex, age, risk factors, hospital level, diagnosis year of HIV infected, and care continuity were used as variables to evaluate the three aforementioned indicators. We used t-tests, chi-square tests and multivariate regression models to examine differences between groups in three long-term viral load indicators, as well as the relationship between the indicators of viral suppression and patient death during study period. We constructed logistic regression to determine the factors associated with durable viral suppression. *P* value < 0.05 was considered statistically significant.

## Results

A total of 27,706 patients were included, and the median number of viral tests were 4. Of whom 95.3% were male, 47.3% were age 20–29 when infected with HIV, 36.1% aged 30–39 in the study, 58.5% with college or higher educational level, 73.0% risk factor of infecting with HIV was men who have sex with men, 40.9% were resided in the Taipei region, 55.3% received treatment at a medical centres, 2.6% categorized as interrupted care, 93.6% categorized as continual care, 81.3% with CD4 count more than 350 cell/mm^3^ at the beginning of this study, 51.1% were reported before 2010, and 5.1% with a history of drug resistance.

### Durable viral suppression (viral load < 200 copies/ml)

24,133(87.1%) of the patients experienced durable viral suppression. Analysis of the demographic variables revealed that, compared with other patients, a significantly lower rate of durable viral suppression were observed in patients who were female, under 20 years of age, had a senior high school degree or lower, whose risk factor was being an IDU, resided in eastern Taiwan, received treatment at local hospitals, experienced interrupted care, lacked continual care, had CD4 levels below 350 cell/mm^3^ at the beginning of this study, reported infection before 2010, and had a history of drug resistance (Table 1).

**Table 1:** Distribution of the three indicators of long-term viral load

Variable				Total										Durable viral suppression (< 200 copies/ml)					*P*-value			Mean number of days with a viral load > 1,500 copies/ml			*P*-value		Geometric mean of viremia copy-years		*P*-value	
				*N*						%				*n*			%					*n*	%	mean days						
Total				27,706						100.0				24,133			87.1					1,468	5.3	184			90			
Sex																				< 0.001					0.23				0.01	
Female				1,304						4.7				1,094			83.9					82	6.3	202			100			
Male				26,402						95.3				23,039			87.3					1,386	5.3	185			89			
Age when infected																				< 0.001					0.32				0.03	
< 20				823						3.0				671			81.5					59	7.2	182			104			
20–29				13,094						47.3				11,227			85.7					790	6.0	189			90			
30–39				9,110						32.9				8,010			87.9					457	5.0	185			81			
40–49				3,405						12.3				3,070			90.2					119	3.5	178			75			
>=50				1,274						4.6				1,155			90.7					59	4.6	150			74			
Age in this study																				< 0.001					0.12			0.02		
< 20						25				0.1				18			72.0					2	8.0	393			160			
20–29						3,208				11.6				2,702			84.2					224	7.0	195			103			
30–39						10,010				36.1				8,700			86.9					568	15.7	186			92			
40–49						8,497				30.7				7,387			86.9					435	5.1	184			90			
>=50						5,966				21.5				5,326			89.3					239	4.0	178			79			
Educational level																				< 0.001					0.37			< 0.001		
Senior high school or lower								11,494		41.5				9,404			81.8					875	7.6	188			110			
College or higher								16,212		58.5				14,729			90.9					593	3.7	182			77			
Risk factor																				< 0.001					0.08			0.05		
MSM								20,230		73.0				17,984			88.9					963	4.8	185			85			
Heterosexual (female)								718		2.6				643			89.6					35	4.9	186			82			
Heterosexual (male)								2,996		10.8				2,675			89.3					136	4.5	168			77			
IDU								3,718		13.4				2,795			75.2					331	8.9	195			138			
Residence																				< 0.001					< 0.001			0.10		
Taipei region								11,333		40.9				10,213			90.1					427	3.8	189			91			
Northern region								3,919		14.1				3,331			85.0					230	5.9	213			106			
Central region								4,611		16.6				3,956			85.8					283	6.1	157			77			
Southern region								2,702		9.8				2,305			85.3					166	6.1	180			110			
Kaohsiung and Pingtung region								4,660		16.8				3,963			85.0					300	6.4	190			74			
Eastern region								481		1.7				365			75.9					62	12.9	195			139			
Hospital level																				< 0.001					< 0.001			0.10		
Medical centre								15,330		55.3				13,746			89.7					695	4.5	169			73			
Regional hospital								11,654		42.1				9,930			85.2					656	5.6	201			110			
Local hospital								566		2.0				392			69.3					66	11.7	172			176			
Clinic								2		0.0				2			0.0					0	0.0	-			65			
Interrupted care																				< 0.001					< 0.001			< 0.001		
No								26,972		97.4				23,702			87.9					1,350	5.0	171			86			
Yes								734		2.6				431			58.7					118	16.1	360			496			
Continual care																				< 0.001					< 0.001			< 0.001		
No								1,778		6.4				747			42.0					358	20.1	251			624			
Yes								25,928		93.6				23,386			90.2					1,110	4.3	165			78			
CD4 level at the beginning of study																				< 0.001					0.001			< 0.001		
< 350								5,161		18.6				3,637			70.5					633	12.3	196			207			
>=350								22,530		81.3				20,484			90.9					835	3.7	178			74			
Reported year																				< 0.001					0.76			0.001		
Before 2010								14,161		51.1				12,182			86.0					768	5.4	185			92			
After 2010								13,545		48.9				11,951			88.2					700	5.2	187			87			
History of drug resistance																				< 0.001					0.49			< 0.001		
No								26,297		94.9				23,101			87.8					1,264	4.8	187			86			
Yes								1,409		5.1				1,032			73.2					204	14.5	180			192			

### Days with a viral load over 1,500 copies/ml

Of all patients, 1,468(5.3%) had a viral load over 1,500 copies/ml for an average of 184 days from 2019 to 2020. Compared with other patients, patients who were female, young, or had a senior high school degree or lower experienced more days with a viral load over 1,500 copies/ml, although the difference wasn’t statistically significant. On the respect of risk factors, IDUs experienced an average of 195 days with a viral load over 1,500 copies/ml, which are greater than those experienced by MSM or heterosexual transmission. Compared with other patients, patients residing in northern Taiwan (213 days), those treated at regional hospitals (201 days), individuals with interrupted care (360 days), those lacking continual care (215 days), and those CD4 levels below 350 at the beginning of this study (196 days), experienced a significantly more days with a viral load over 1,500 copies/ml (Table 1).

### Viremia copy-years

Table 1 shows the geometric mean of viremia copy-years. For all patients, the mean of viremia was 90 copy*years/ml, indicating that their mean viral replication was 90 copies each year. A larger number of copies indicated greater viral replication and a higher risk of infection. Compared with other patients, females (100 copy*years/ml), those under 20 years of age (104 copy*years/ml), individuals with a senior high school degree or lower (110 copy*years/ml), those risk factor was being an IDU (138 copy*years/ml), those with interrupted care (496 copy*years/ml), those lacking continual care (624 copy*years/ml), individuals with CD4 levels below 350 at the beginning of this study (207 copy*years/ml), those whose infection was reported before 2010 (92 copy*years/ml), those with a history of drug resistance (192 copy*years/ml) had significantly higher viremia copy-years, particularly for those lacking continual care (624 copy*years/ml) and those with interrupted care (496 copy*years/ml).

This study compared the relative differences between patients’ final viral load test results and durable viral suppression. Regardless of demographic variables, the proportion of patients experiencing durable viral suppression was consistently lower than those experiencing viral suppression based on the final viral load test, with a 9.3% relative difference. Specifically, viral suppression levels were overestimated for specific groups: IDUs (19.4%), those treated at local hospitals (25.6%), those with interrupted care (23.6%), those lacking continual care (44.4%), and those with CD4 levels below 350 at the study’s onset (22.3%) (Table 2).

**Table 2:** Difference between viral suppression of patients sampled at a single time point and durable viral suppression

Variable	Total			Final viral load test (< 200 copies/ml) (A)			Durable viral suppression (< 200 copies/ml) (B)			Relative difference
	*N*	%		*n*	%		*n*	%		$\left(\frac{(B-A)}{A}\right)\times 100$
Total	27,706	100.0		26,602	96.0		24,133	87.1		-9.3
Sex										
Female	1,304	4.7		1,227	4.6		1,094	4.5		-10.8
Male	26,402	95.3		25,375	95.4		23,039	95.5		-9.2
Age when infection was reported										
<20	823	3.0		768	2.9		671	2.8		-12.6
20–29	13,094	47.3		12,479	46.9		11,227	46.5		-10.0
30–39	9,110	32.9		8,800	33.1		8,010	33.2		-9.0
40–49	3,405	12.3		3,317	12.5		3,070	12.7		-7.4
>=50	1,274	4.6		1,238	4.7		1,155	4.8		-6.7
Age in this study										
<20	25	0.1		20	0.1		18	0.1		-10.0
20–29	3,208	11.6		3,043	11.4		2,702	11.2		-11.2
30–39	10,010	36.1		9,582	36.0		8,700	36.1		-9.2
40–49	8,497	30.7		8,159	30.7		7,387	30.6		-9.5
>=50	5,966	21.5		5,798	21.8		5,326	22.1		-8.1
Educational level										
Senior high school or lower	11,494	41.5		10,838	40.7		9,404	39.0		-13.2
College or higher	16,212	58.5		15,764	59.3		14,729	61.0		-6.6
Risk factor										
MSM	20,230	73.0		19,513	73.4		17,984	74.5		-7.8
Heterosexual (female)	718	2.6		690	2.6		643	2.7		-6.8
Heterosexual (male)	2,996	10.8		2,894	10.9		2,675	11.1		-7.6
IDU	3,718	13.4		3,466	13.0		2,795	11.6		-19.4
Other factors	44	0.2		39	0.1		36	0.1		-7.7
Residence										
Taipei region	11,333	40.9		10,988	41.3		10,213	42.3		-7.1
Northern region	3,919	14.1		3,747	14.1		3,331	13.8		-11.1
Central region	4,611	16.6		4,412	16.6		3,956	16.4		-10.3
Southern region	2,702	9.8		2,583	9.7		2,305	9.6		-10.8
Kaohsiung and Pingtung region	4,660	16.8		4,443	16.7		3,963	16.4		-10.8
Eastern region	481	1.7		429	1.6		365	1.5		-14.9
Hospital level										
Medical centre	15,330	55.3		14,839	55.8		13,746	57.0		-7.4
Regional hospital	11,654	42.1		11,157	41.9		9,930	41.1		-11.0
Local hospital	566	2.0		527	2.0		392	1.6		-25.6
Clinic	2	0.0		2	0.0		2	0.0		0.0
Interrupted care										
No	26,972	97.4		26,038	97.9		23,702	98.2		-9.0
Yes	734	2.6		564	2.1		431	1.8		-23.6
Continual care										
No	1,778	6.4		1,344	5.1		747	3.1		-44.4
Yes	25,928	93.6		25,258	94.9		23,386	96.9		-7.4
CD4 at the beginning of this study										
< 350	5,161	18.6		4,680	17.6		3,637	15.1		-22.3
>=350	22,530	81.3		21,909	82.4		20,484	84.9		-6.5
Year in which infection was reported										
Before 2010	14,161	51.1		13,565	51.0		12,182	50.5		-10.2
After 2010	13,545	48.9		13,037	49.0		11,951	49.5		-8.3
History of drug resistance										
No	26,297	94.9		25,334	95.2		23,101	95.7		-8.8
Yes	1,409	5.1		1,268	4.8		1,032	4.3		-18.6

Factors associated with durable viral suppression were examined (Table 3). Patients under 50 years of age, with an education level of senior high school or lower, and with IDU risk factors were more likely not to experience durable viral suppression. Additionally, patients living outside Taipei, particularly in eastern Taiwan, were at higher risk. Those treated at regional or local hospitals were more likely not to experience durable viral suppression than those at medical centers. Continual care significantly reduced the likelihood of not experiencing durable viral suppression. Patients with CD4 levels exceeding 350 at the study’s beginning were more likely to achieve durable viral suppression (Table 3).

**Table 3:** Factors affecting durable viral suppression

Variable	*N* = 27,706	OR	95% CI	*p*-value		Adjust OR (stepwise)	95% CI	*p*-value
Sex (ref. male)								
Female	26,402	1.3	1.1 ~ 1.5	0.0004		1.1	0.9 ~ 1.3	0.3893
Age (ref. > 50)								
< 20	6,564	2.2	1.7 ~ 2.8	< 0.0001		2.9	2.2 ~ 3.9	< 0.0001
20–29	12,938	1.6	1.3 ~ 1.9	< 0.0001		2.3	1.9 ~ 2.9	< 0.0001
30–39	5,718	1.3	1.1 ~ 1.6	0.0104		1.7	1.4 ~ 2.1	< 0.0001
40–49	1,868	1	0.8 ~ 1.3	0.8294		1.2	0.9 ~ 1.5	0.208
Educational level (ref. college or higher)								
Senior high school or lower	11,494	2.2	2.0 ~ 2.4	< 0.0001		1.7	1.6 ~ 1.9	< 0.0001
Risk factor (ref. MSM)								
IDU	3,718	2.6	2.4 ~ 2.8	< 0.0001		1.2	1.1 ~ 1.4	0.0018
Heterosexual	3,714	1	0.9 ~ 1.1	0.3725		1	0.9 ~ 1.1	0.8274
Other factors	44	1.6	0.7 ~ 3.7	0.2392		0.8	0.3 ~ 2.0	0.5677
Residence (ref. Taipei region)								
Northern region	3,919	1.6	1.5 ~ 1.8	< 0.0001		1.2	1.1 ~ 1.4	0.0024
Central region	4,611	1.5	1.4 ~ 1.7	< 0.0001		1.5	1.3 ~ 1.7	< 0.0001
Southern region	2,702	1.6	1.4 ~ 1.8	< 0.0001		1.3	1.2 ~ 1.5	< 0.0001
Kaohsiung and Pingtung region	4,660	1.6	1.5 ~ 1.8	< 0.0001		1.4	1.2 ~ 1.6	< 0.0001
Eastern region	481	3	2.4 ~ 3.7	< 0.0001		2.6	2.0 ~ 3.3	< 0.0001
Hospital level (ref. medical centre)								
Regional hospital	11,654	1.5	1.4 ~ 1.6	< 0.0001		1.4	1.3 ~ 1.5	< 0.0001
Local hospital	566	3.9	3.2 ~ 4.6	< 0.0001		2.3	1.9 ~ 2.9	< 0.0001
Continual care (ref. No)								
Yes	25,928	0.1	0.1 ~ 0.1	< 0.0001		0.1	0.1 ~ 0.1	< 0.0001
CD4 level at the beginning of this study (ref. <350)								
> 350	22,530	0.2	0.2 ~ 0.3	< 0.0001		0.3	0.3 ~ 0.3	< 0.0001

The study explored the link between long-term viral suppression indicators and patient mortality (Table 4). Patients who died within the study period showed a higher proportion of a final viral load exceeding 200 copies/ml compared to survivors (3.5% vs. 0.8%). The proportion of patients with durable viral suppression who died during the study was significantly smaller than those who survived (2.2% vs. 0.7%). Moreover, the number of viremia copy-years for those who died was larger than for survivors (126 copy years/ml vs. 89 copy years/ml).

**Table 4:** Relationship between the indicators of viral suppression and patient death

Variable	Total (*n*)		Subjects who died within the study period			Subjects who did not die within the study period		*p*-value
			*n*	%		*n*	%	
Total	27,706		238	0.9		27,468	99.1	
Final viral load < 200 copies/ml								< 0.0001
No	1,104		39	3.5		1,065	96.5	
Yes	26,602		199	0.7		26,403	99.3	
Durable viral suppression < 200 copies/ml								< 0.0001
No	3,573		79	2.2		3,494	97.8	
Yes	24,133		159	0.7		23,974	99.3	
			Number of days			Number of days		
Mean number of days with a viral load > 1,500 copies/ml	1,468		135			186		0.0098
			copy*years/mL			copy*years/mL		
Geometric mean of viremia copy-years	27,706		126			89		0.0225

## Discussion

According to the study results, 87.1% of the patients included in this study experienced durable viral suppression, of whom 5.3% had a viral load exceeding 1,500 copies/ml for an average of 184 days, with a mean number of viremia copy-years of 90 copy*years/ml. To obtain further insights, the relationship between viral load indicators, which relied on a final viral load smaller than 200 copies/ml to identify viral suppression, and three indicators of long-term viral load was investigated. The results indicated that when final viral load tests were conducted from 2019 to 2020, 96% of the patients experienced viral suppression, with only 87.1% experiencing durable viral suppression. These results indicated that conducting a single viral load test to determine viral suppression resulted in an overestimation of viral suppression, with a relative difference of 9.3% between the two indicators. In addition, 4.0% of the patients whose final viral load was reported to be less than 200 copies/ml (*n* = 1,066) actually exhibited a viral load of over 1,500 copies/ml, and 1.7% of them (*n* = 442) had a high viral load for over 6 months. These results indicated that conducting a single viral load test resulted in an overestimation of viral load. These findings are consistent with those of other international studies [20–22]. Patients whose risk factor was being an IDU, who received treatment at local hospitals, who had interrupted care, who did not receive continual care, and whose CD4 levels were below 350 at the beginning of this study had overestimated viral suppression.

Compared with other patients, patients who were female, who were under 20 years of age, whose risk factor was being an IDU, who resided in eastern Taiwan, who received treatment at local hospitals, who had interrupted care, and whose CD4 levels were below 350 at the beginning of this study experienced lower durable viral suppression. Further analyses were conducted to determine why patients who received treatment at local hospitals experienced low durable viral suppression. The results indicated that the majority of patients whose risk factor was being an IDU visited local hospitals to receive treatment. As a result of their drug use patterns, IDUs typically receive irregular treatment, and they exhibit poor compliance towards treatment [23]. These factors may be the reason underlying why patients who received treatment at local hospitals experienced low durable viral suppression. In addition, the majority of patients aged 20–29 received treatment at local hospitals. Generally, younger individuals exhibit poorer compliance towards treatment compared with other age groups. This phenomenon may have affected the viral suppression levels of patients who received treatment at local hospitals.

The relationship between the three indicators of long-term viral load and patient death was investigated. The results indicated that, patients whose final viral load exceeded 200 copies/ml, who did not experience durable viral suppression, and who experienced a large number of viremia copy-years had higher mortality rates. Further research is required to examine the relationship between single or long-term indicators of viral suppression and patient death.

This study has five limitations. First, the patients included in this study underwent at least one viral load test in 2018 and at least two viral load tests in 2019 and 2020. This inclusion criterion, requiring a minimum of three medical visits, may result in selection bias by excluding individuals who never sought medical care, irregular attendees, or those who passed away during 2018–2021. Additionally, patients not receiving treatment or lacking data for 2019–2020 were excluded, further influencing the generalizability of durable viral suppression results. Therefore, the overall durable viral suppression levels may have been overestimated. Second, only 1,409 patients (5%) had a history of drug resistance. Those without a history of drug resistance either actually had no history of drug resistance or just never had their drug resistance levels tested. In addition, patients who had a history of drug resistance were not compared with those who received continual care. Because continual care is associated with drug resistance, some patients may have developed drug resistance and required additional continual care, or they may have developed drug resistance because they did not receive continual care. This study was unable to verify the relationship between drug resistance and long-term viral suppression. Third, this study included patients who underwent two or more viral load tests in 2019 and 2020. Generally, undergoing a viral load test does not indicate whether a patient has excellent compliance towards treatment. Some patients may undergo a viral load test but still not take their medications. Therefore, the status of their treatment cannot be verified. Future studies should determine the effect of medication compliance on viral suppression. Fourth, patients who reported their infection in 2018 may have reported lower viral suppression levels compared with other patients because they have been taking their medications for a considerably shorter time. The current policy is to provide patients with their medications as soon as they receive their diagnoses, with over 90% of all patients usually receiving their medications within 1 month of diagnosis. Therefore, although the effect of medication on long-term viral load may not be adequate, viral load changes should still be monitored in patients. On the fifth, this study only evaluated data from two years after treatment, and treatment failure may occur more than two years later, the follow-up time may not be long enough in this study may affect the extrapolation of test results.

## Conclusion

According to the study results, conducting a final viral load test to determine the viral suppression levels of patients may result in an overestimation of viral suppression. Long-term monitoring of viral load may help reveal cumulative viral replication over time, and data acquired in this manner may serve as an indicator of case management or healthcare quality. Therefore, viral suppression should be identified both by indicators adopted at a single time point and by long-term monitoring to precisely track viral load changes in patients, immediately revise case management strategies, and mitigate the risk of HIV transmission.

## Data Availability

The data that support the findings of this study are available from the corresponding author upon reasonable request.
